# Liver Transplant Complications Radiologist Can't Miss

**DOI:** 10.7759/cureus.8465

**Published:** 2020-06-05

**Authors:** Yukiyoshi Kimura, Ramiro Tapia Sosa, Dafne Soto-Trujillo, Yumi Kimura Sandoval, Carlos Casian

**Affiliations:** 1 Radiology, Instituto Nacional De Ciencias Medicas Y Nutricion Salvador Zubiran, Mexico City, MEX; 2 Gastroenterology, Instituto Nacional De Ciencias Medicas Y Nutricion Salvador Zubiran, Mexico City, MEX; 3 Radiology, Centro Medico ABC, Mexico City, MEX

**Keywords:** hepatic transplant, hepatic transplant complications, radiology, interventional radiology

## Abstract

Liver transplantation is considered the ideal and definitive therapy for patients with end-stage liver disease. Since 1963 advances in liver transplant surgical techniques and immunosuppressive therapies have improved outcomes and patients' survival. However, early diagnosis of graft dysfunction, through different imaging modalities, is crucial for graft survival. Imaging plays a fundamental role before, during and after the transplantation process. In this review, we will discuss the importance of imaging in the diagnosis of vascular and biliary post-transplant complications through different imaging modalities such as Doppler ultrasonography, computed tomography (CT) and magnetic resonance imaging (MRI).

## Introduction and background

Orthotopic liver transplant is the ideal and definitive treatment for patients with end-stage acute liver failure or chronic liver disease [1].

The first liver transplant was performed by Starzl et al. in 1963 [2]. Since then advances in surgical techniques and immunosuppressive therapies have significantly improved outcomes [3,4]. Each year more than 6,000 liver transplant procedures are performed in the United States. However, organ shortage increases year after year, with more than 16,865 patients on the waiting list in 2011 [5].

Liver transplant indications

The liver transplant must be considered in any patient with end-stage liver disease, in whom life expectancy would be extended beyond the natural history of the underlying disease [6].

Following are the indications:

- Decompensated cirrhosis (fulminant hepatic failure / encephalopathy / ascites / variceal hemorrhage / hepatocellular carcinoma (Milan))

- Hepatitis (End-stage liver disease) (HBV / HCV / Autoimmune)

- Chronic cholestatic diseases (primary biliary cirrhosis / primary sclerosing cholangitis)

- Metabolic hepatic diseases (hemochromatosis / Wilson’s disease) [7].

The Child-Pugh-Turcotte classification and the Model of End-stage Liver Disease (MELD, MELD-Na and Delta MELD) are used for patient priority assessment [6]. MELD predicts three-month mortality in patients with end-stage liver disease [8].

Merion et al. compared relative mortality rates between deceased liver transplant recipients and candidates among several MELD score groups. In their study, they found that mortality risk was 38% lower in recipients compared to candidates in the MELD 18-20 group. As the MELD score increased, survival benefit significantly increased. Mortality risk was 96% lower in transplanted patients with the highest MELD score (40) than in candidates. However, recipient mortality risk during the first post-transplant year increased with MELD scores lower than 15 compared to candidates [8].

The table below (Table 1) shows the hazard ratio for post-transplant mortality risk based on MELD score at transplant, compared to wait-listing at comparable MELD score groups [8].

**Table 1:** Hazard ratio for post-transplant mortality risk based on MELD score at transplant, compared to wait-listing at comparable MELD score groups HR: Hazard Ratio; MELD: Model of End-stage Liver Disease.

MELD score group	0-7 days (HR)	8-30 days (HR)	31-365 days (HR)
6-11	33	9	1.8
12-14	28	1.2	1.8
15-17	5.9	2.5	0.8
18-20	2.8	1.13	0.5
21-29	1.6	0.5	0.15
30-39	0.2	0.09	0.06
40	0.11	0.04	0.02

A MELD score higher than 14 is recommended to list patients with end-stage liver disease [6].

The most common causes of primary diseases leading to liver transplantation in Europe (1998-2011) are listed below (Table 2) [6].

**Table 2:** Causes of liver transplantation *Others (Budd–Chiari, benign liver tumors, parasitic disease)

Causes of liver transplantation	Percentage
Cirrhosis	57%
Cancer	15%
Cholestatic Disease	10%
Acute Hepatic Failure	8%
Metabolic Disease	6%
Others*	4%

Liver transplant contraindications

Liver transplant contraindications are dynamic and change between transplant centers and over time.

Some proposed contraindications at different centers are listed below [6,8]:

- Acute extrahepatic malignancy (glioblastoma multiforme, melanoma, choriocarcinoma, lung cancer, amongst others)

- Diffuse hepatic tumoral disease

- Diffuse portal thrombosis (smv)

- Active / uncontrolled systemic infection

- Severe cardiopulmonary disease

- Poor social support

- Poor adherence to treatment

Surgical techniques

Understanding of surgical techniques is crucial to comprehend post-surgical anatomic changes and imaging evaluation. Orthotopic implantation of deceased donor grafts is most commonly used in liver transplants; however, split or reduced deceased donor allografts (pediatric population) and living donor segmental liver transplantation are other options.

**Figure 1:**
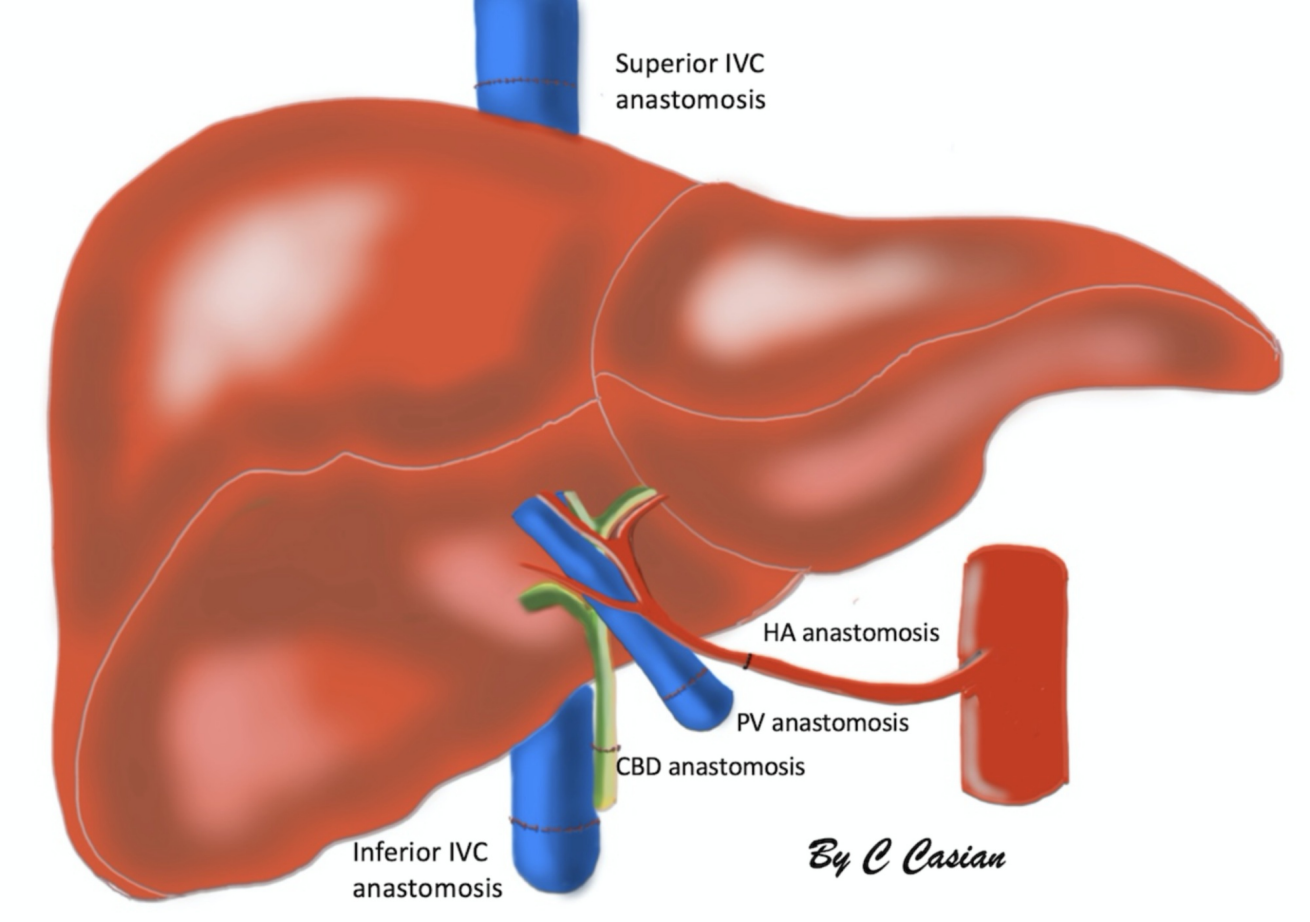
Common vascular and biliary anastomosis with an inferior vena cava (IVC) graft interposition.

**Figure 2:**
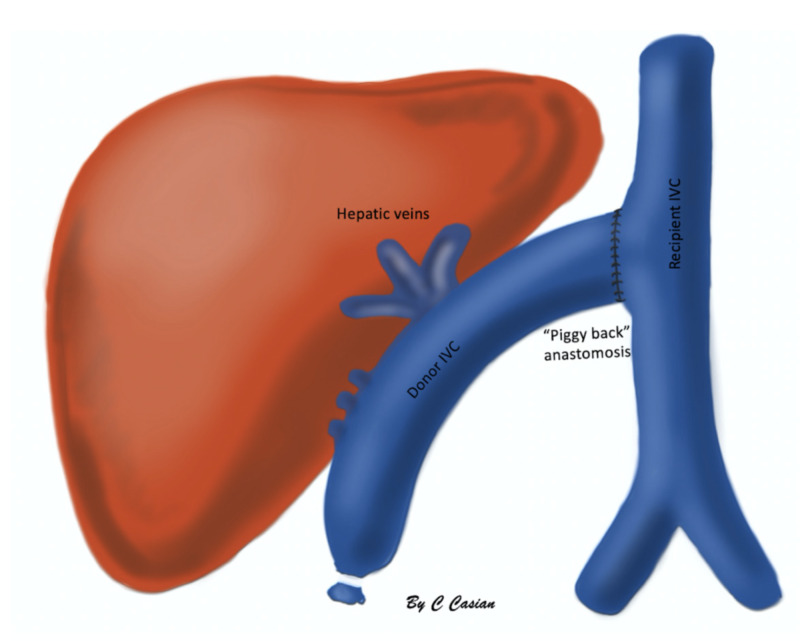
A "piggy back" anastomosis of the inferior vena cava (IVC) as an alternative surgical technique, in which venous circulation is not interrupted.

Three vascular (hepatic artery, portal vein, and inferior vena cava) and a biliary anastomosis are performed (Figures 1, 2).

**Figure 3:**
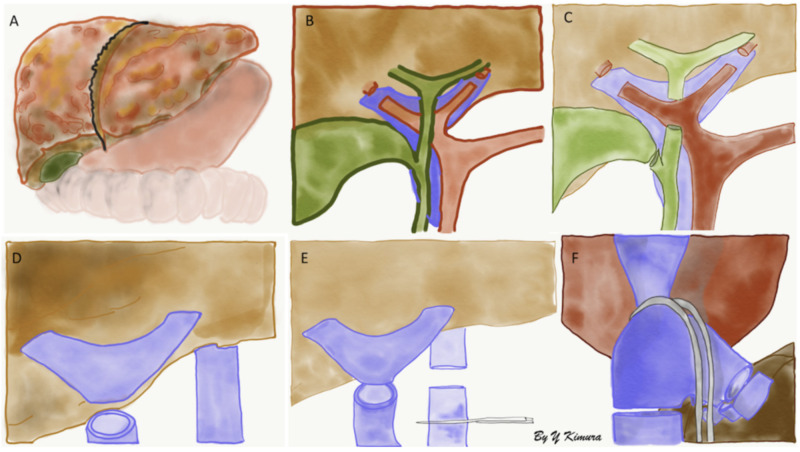
A) Deceased liver from recipient, B) division of the right and left hepatic arteries, C) common hepatic and cystic ducts are divided, D) division of portal vein, E & F) clamps are applied to inferior vena cava (IVC) (below (E) and above (F) the liver) and hepatic veins and IVC are divided.

**Figure 4:**
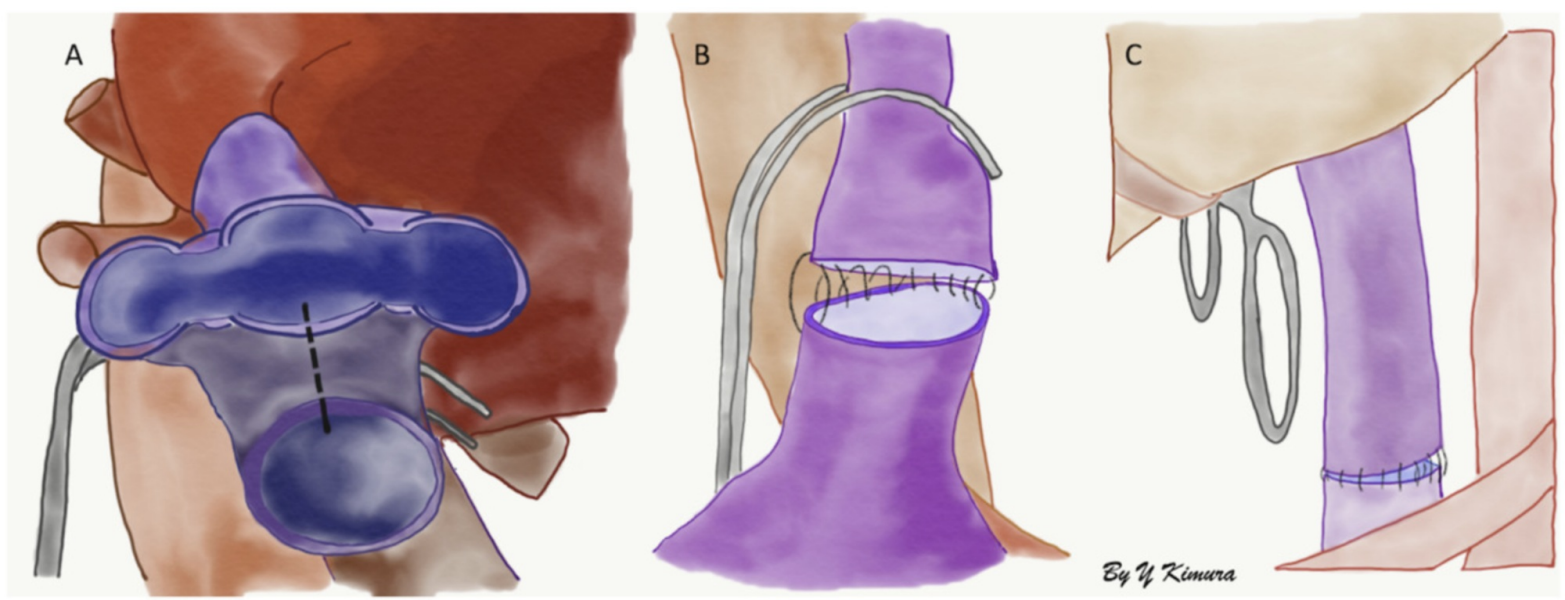
A) Creation of a common vessel between the recipient's three hepatic veins and proximal inferior vena cava (IVC), B) suprahepatic end-to-end anastomosis between the recipient common vessel and donor's IVC. C) Infrahepatic end-to-end anastomosis between the recipient IVC and donor's IVC.

**Figure 5:**
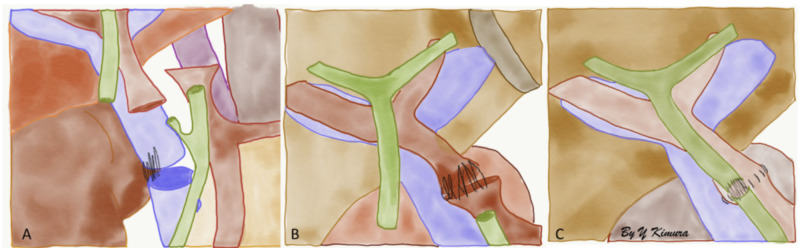
A) Portal vein anastomosis between donor and recipient's portal vein, B) hepatic artery anastomosis, most commonly performed end-to-end anastomosis, between a Carrel patch (rim of deceased donor aortic wall) and the origin of the gastroduodenal artery of the recipient. C) Biliary end-to-end anastomosis between the donor's common bile duct and recipient's common hepatic duct.

Surgical technique with inferior vena cava graft interposition is represented in the figures below (Figures 3-5).

The role of imaging in hepatic transplant

Diagnostic imaging plays an important role in pre-operative evaluation (hepatic graft and recipient selection) and patient follow-up. Early diagnosis of complications is vital for their successful management and graft survival. Clinical signs may vary and are non-specific. Most diagnoses are based on imaging studies, except for graft rejection, which is diagnosed by graft biopsy and histological analysis.

The main role of non-invasive imaging studies is to identify vascular, biliary and parenchymal complications.

Doppler ultrasonography is the preferred post-operative screening imaging modality because it is accessible, cost-effective and can be performed at the bedside. A screening ultrasound is highly recommended within the first 24 hours post-surgery [4].

## Review

Normal postoperative ultrasonographic findings

**Figure 6:**
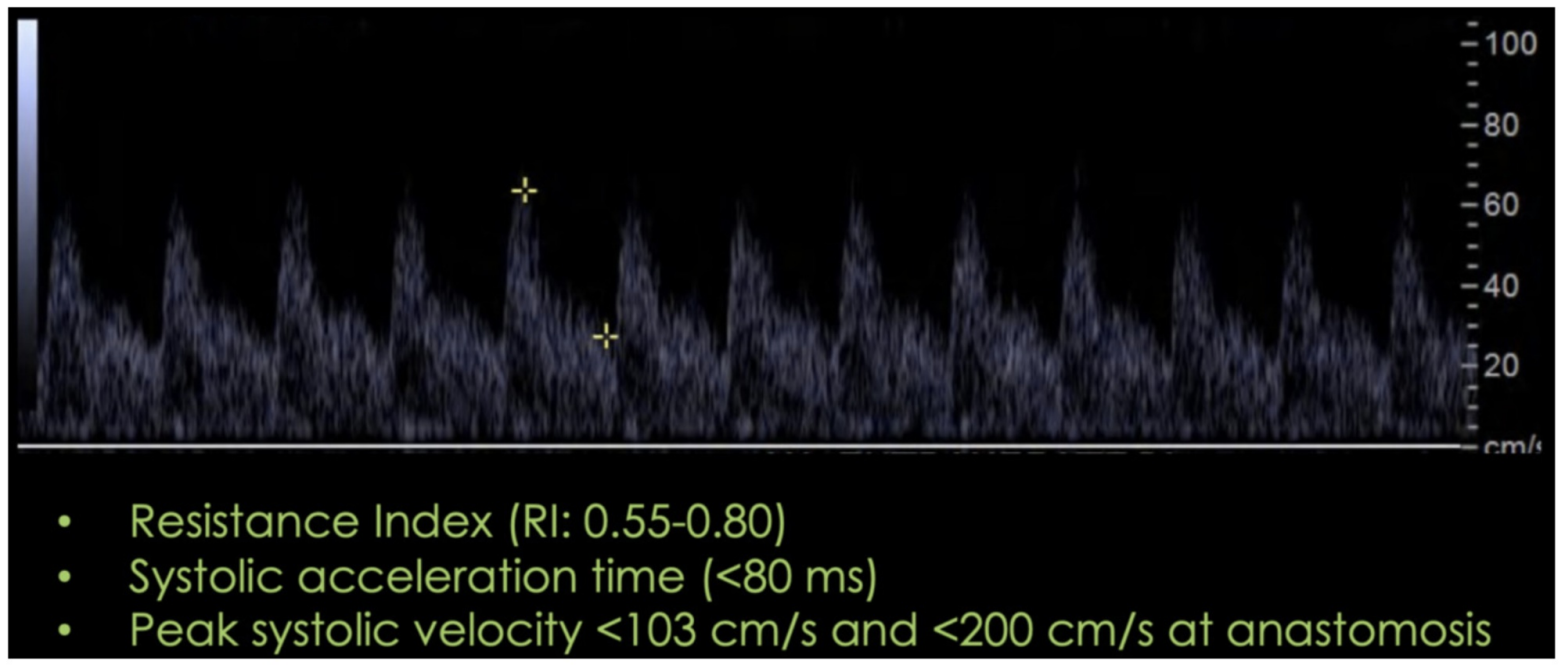
Expected post-surgical spectral waveform in the hepatic artery.

The hepatic artery has a rapid systolic upstroke with a resistance index in the range of 0.5 to 0.8, and an acceleration time under 0.08 seconds [9]. Peak systolic velocities at the hepatic artery anastomosis should not be greater than 200 cm/s (see Figure 6). Inappropriate Doppler angle correction (should not be greater than 60 degrees) and vessel tortuosity falsely elevate velocities.

A resistance index (RI) higher than 0.80 in the immediate post-operative period is not associated with graft complications and is attributed to re-perfusion edema, prolonged cold ischemia time, or vessel spasm, which increases hepatic vascular resistance and decreases compliance [9]. However, the RI must normalize after 7-15 days. Close follow-up with Doppler ultrasound must be performed.

An absent diastolic flow in a previously normal arterial spectral waveform raises concern for an impending arterial complication (thrombosis) and further imaging (CT/MR angiography) is highly encouraged.

On the other hand, an increased diastolic velocity with a low resistance index (below 0.6) is an ominous finding, with a 100% sensibility and 80% specificity for short-term vascular complications [10].

**Figure 7:**
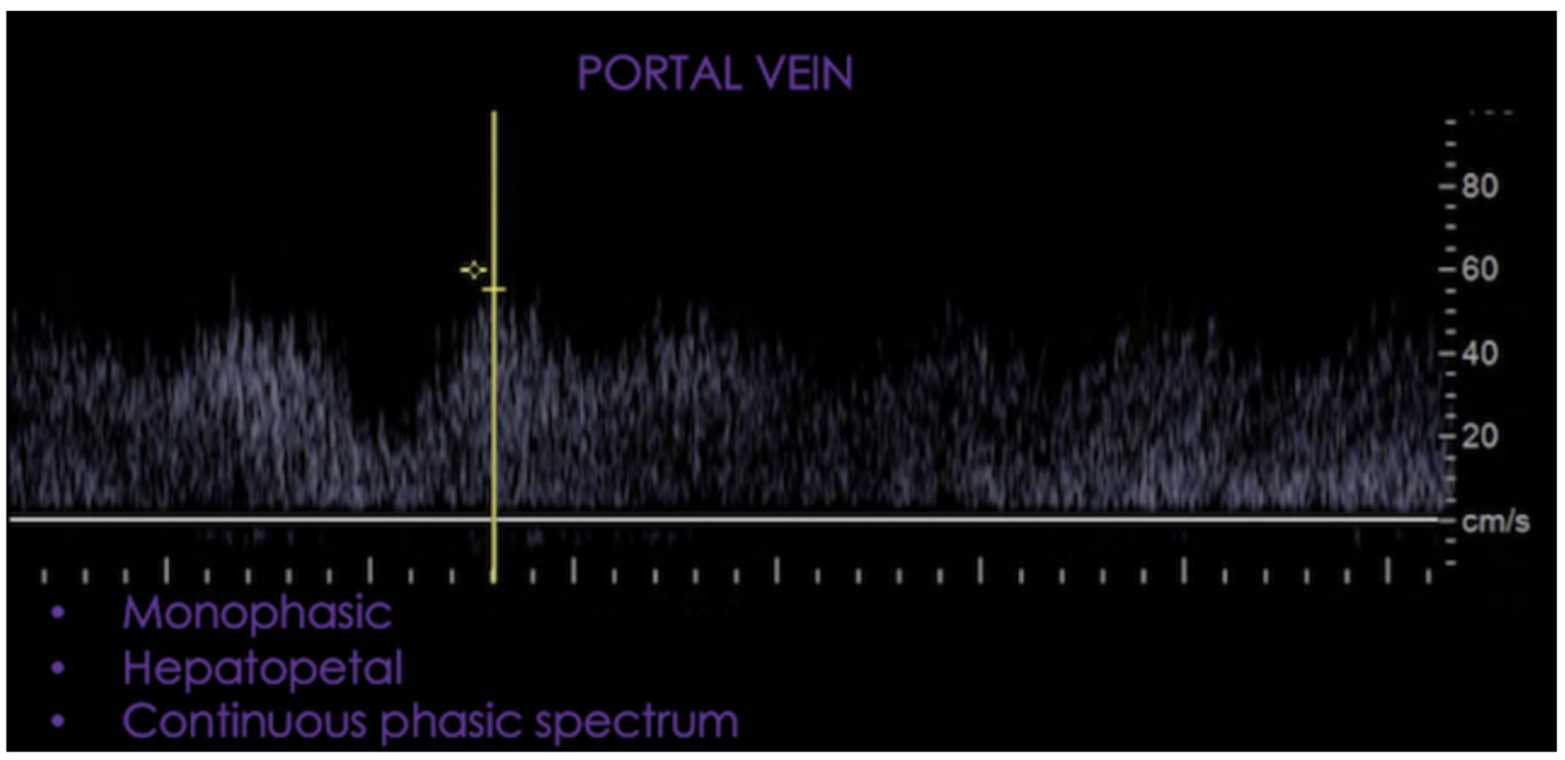
Monophasic, hepatopetal and continuous spectral waveform.

The portal vein has a continuous monophasic and hepatopetal spectral waveform. High portal venous velocities may be seen in the early postsurgical period due to a reduction in portal venous resistance and an increase in portal venous flow, or due to extrinsic compression from a postoperative collection. Velocities should normalize on follow-up studies as the body hemodynamically adapts or as the postoperative collection resolves (see Figure 7).

**Figure 8:**
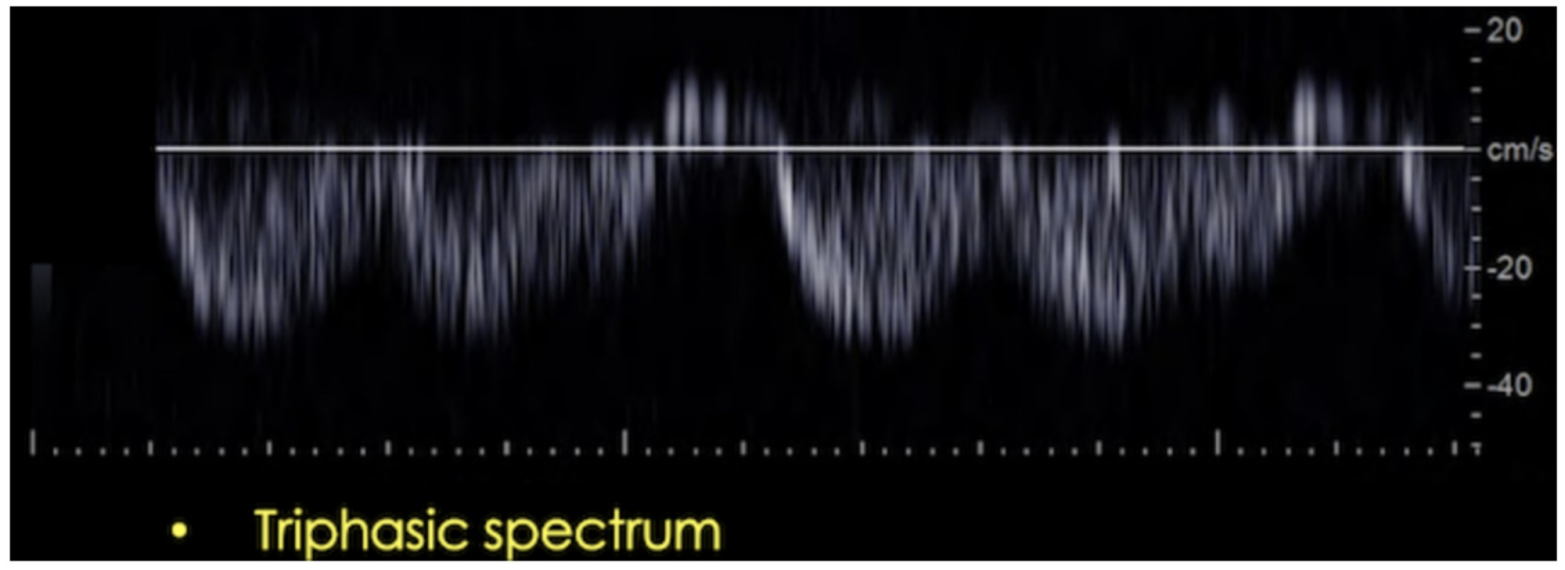
Triphasic spectral waveform in a post-transplant patient.

The hepatic veins and inferior vena cava have a triphasic and anterograde spectrum. Monophasic spectrums are usually secondary to extrinsic compressions but may also be seen in proximal stenosis and vein thrombosis. In patients with tricuspid regurgitation, right heart failure, and fluid overload, a transient monophasic waveform is commonly seen (see Figure 8).

Vascular complications

Vascular complications have an overall incidence of 9%, and in most cases are related to the hepatic artery. They should always be considered in post-transplanted patients who present with hepatic failure, bile leakage, sepsis, or gastrointestinal bleeding [11,12].

Hepatic artery complications

**Figure 9:**
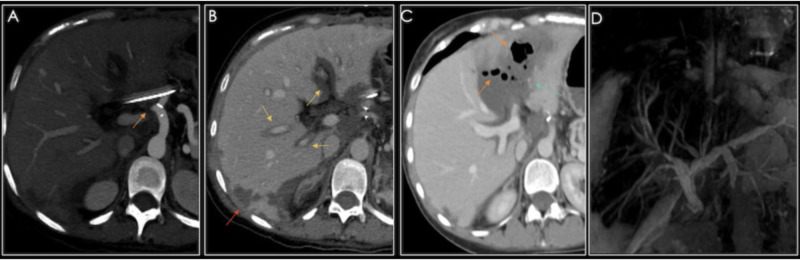
A 24-year-old male with a history of orthotopic liver transplant due to cryptogenic cirrhosis, presenting with abnormal liver function tests. A) Maximum intensity projection showing an abrupt amputation at the origin of the hepatic artery (orange arrow). B) Periportal edema (yellow arrows) and a wedge-shaped zone of peripheral hypoperfusion consistent with an infarction (red arrow), no hepatic arteries are visualized. C) Multiphasic CT showing extensive biliary necrosis with cavitation (orange arrows), suggestive of infection and liquefaction. D) MR cholangiography showing multiple sites of stenosis and irregular intrahepatic biliary dilation.

Hepatic artery complications include hepatic artery thrombosis, stenosis, and pseudoaneurysm. Biliary ducts are exclusively irrigated by branches of the hepatic artery after transplantation; therefore, any arterial complication can cause biliary ischemia (which is an unusual complication in non-transplanted patients due to collateral circulation), strictures, necrosis, and abscess formation (see Figure 9).

Hepatic artery thrombosis

Hepatic artery thrombosis (HAT) is the most common and feared vascular complication, with an estimated incidence of 2-12% in adult recipients and 42% in children [11,13]. It occurs between 15-132 days after surgery, most often within six weeks [9,14].

Risk factors:

- Allograft acute rejection

- ABO blood type incompatibility

- A significant size difference between donor and recipient’s hepatic artery

- Previous stenotic lesion of the celiac axis

- Cytomegalovirus infection

- Pediatric population

- Prolonged cold ischemia time

Doppler ultrasound is a reliable non-invasive imaging technique for evaluating hepatic artery patency (reported sensitivity up to 92%), being able to demonstrate an absence of flow within the vessel and its intrahepatic branches [9]. Decreased diastolic flow and peak systolic velocities are predictors of imminent hepatic artery thrombosis. False-negative interpretations may occur when distal intrahepatic arterial waveforms are seen with a “tardus et parvus” spectrum, through collateral irrigation in chronic thrombosis, simulating arterial stenosis [7,10]. On the other hand, high-grade stenosis, hepatic edema, and hypotension may lead to false-positive interpretations. Contrast-enhanced ultrasound has shown sensitivities and specificities close to 100% [15].

**Figure 10:**
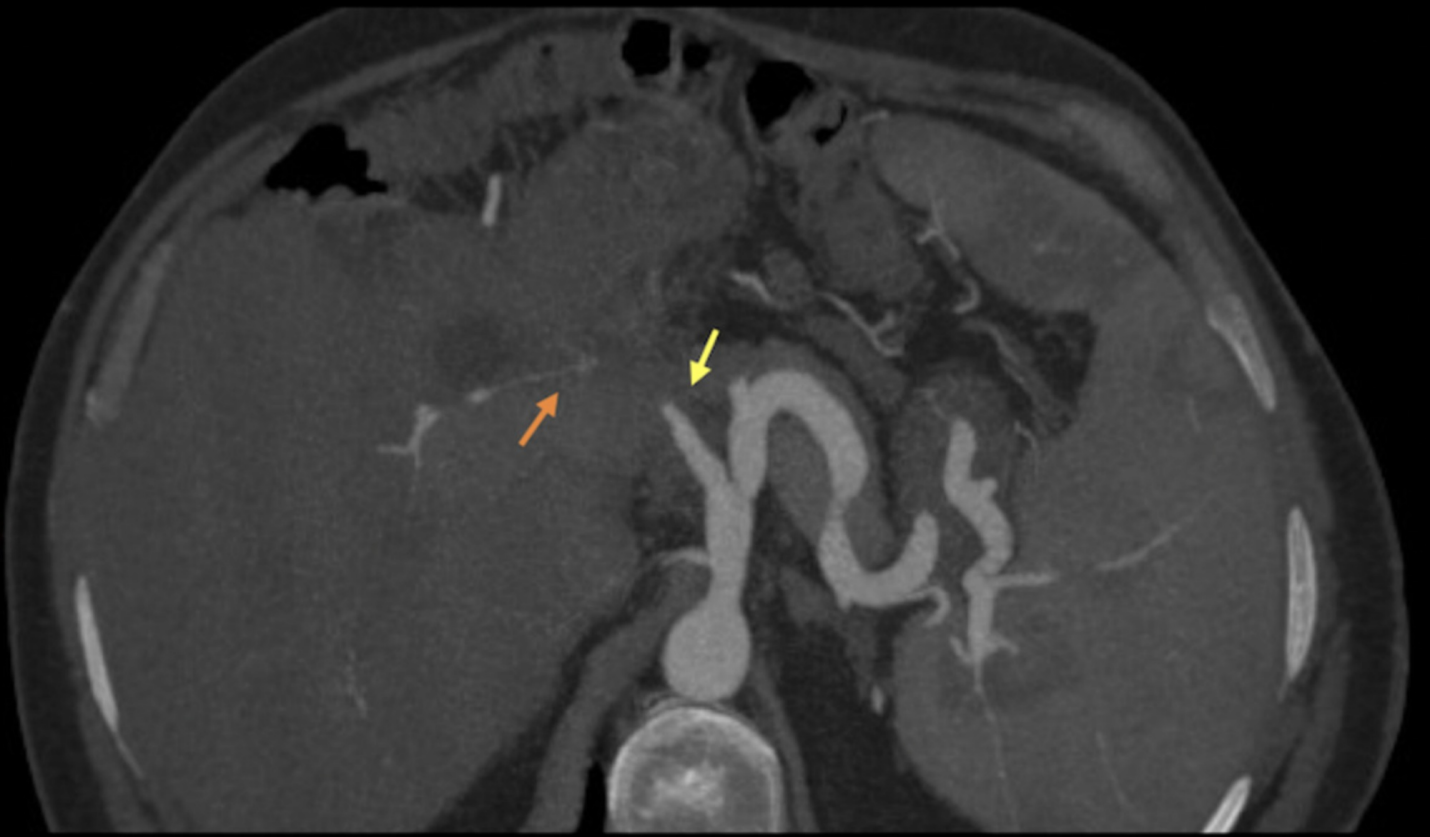
CT angiography maximum intensity projection, showing an amputated hepatic artery (yellow arrow) and opacified intrahepatic arteries due to collateral circulation (orange arrow).

When ultrasonographic findings are positive, computed tomography angiography (CTA) or digital subtraction angiography (DSA) is performed to confirm the diagnosis, in which a filling defect or amputated arterial vessel (most commonly at the anastomosis site) is diagnostic (see Figure 10).

Hepatic artery stenosis

Hepatic artery stenosis (HAS) has an estimated incidence of 2-11% [9]. It often occurs within the first three months following surgery, with a median time of 100 days [11,13].

Risk factors:

- Clamp injury

- Vasa vasorum disruption

- Intimal trauma

**Figure 11:**
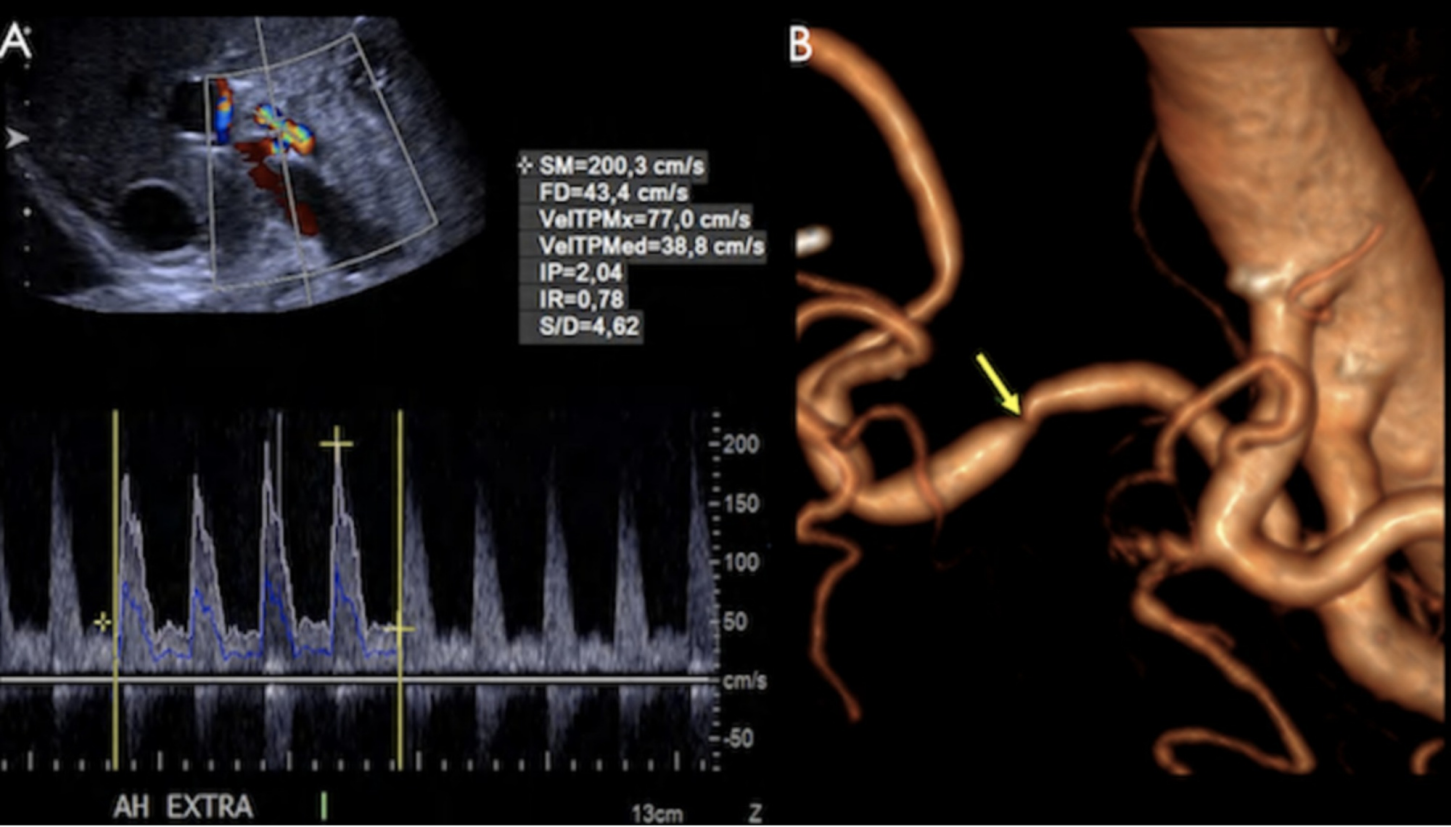
A) Triplex US showing an aliasing hepatic artery. Spectrum: Increased velocities and turbulent flow. B) Volume rendered image from a CT angiography showing stenosis at the site of hepatic artery anastomosis (yellow arrow), with post- stenotic dilation.

Color-Doppler ultrasound is the initial method of choice. Direct findings at the anastomosis site are increased peak systolic velocities (>200 cm/s) (reported positive predictive value of 96%) and turbulent flow (see Figure 11) [9].

**Figure 12:**
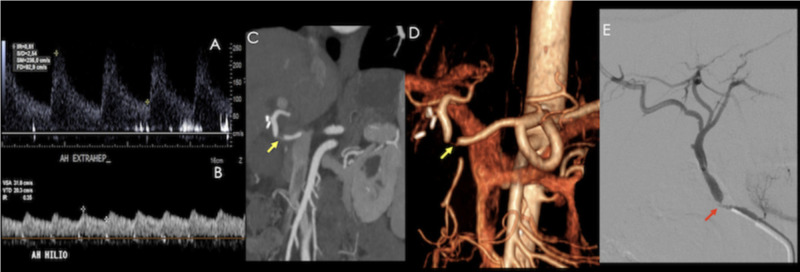
Patient with a history of orthotopic liver transplant presenting with abnormal liver function tests. A) Hepatic artery spectral analysis (at an extrahepatic level) showing increased peak systolic velocity (236 cm/s). B) At the hilum, the spectrum demonstrates a "tardus et parvus" morphology (RI 0.35, with prolonged acceleration time). A CT angiography was performed: C) Coronal reconstruction, D) Volume rendered image, which showed a focal zone of decreased caliber (yellow arrow). E) Confirmed site of stenosis on angiography (red arrow).

Indirect findings downstream from the site of stenosis are an increase in diastolic flow, which decreases the resistance index to less than 0.55-0.5, and a delayed systolic upstroke (peak systolic acceleration time >80 ms), which produces the characteristic “tardus et parvus” spectral waveform of hemodynamically significant stenosis. Indirect findings have a reported sensitivity and specificity of 73-83% and 60-73%, respectively (see Figure 12) [4].

**Figure 13:**
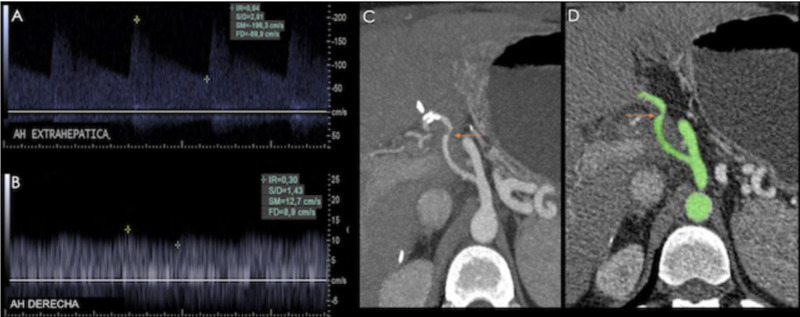
37-year-old male with a history of liver transplant due to cryptogenic cirrhosis, presenting with elevated transaminases and direct hyperbilirubinemia. A) Triplex ultrasound (US) showing an increased peak systolic velocity at the extrahepatic portion of the hepatic artery; B) the spectrum acquires a "tardus et parvus" morphology in its intrahepatic portion. C) & D) CT angiography confirming a focal site of stenosis (orange arrows).

**Figure 14:**
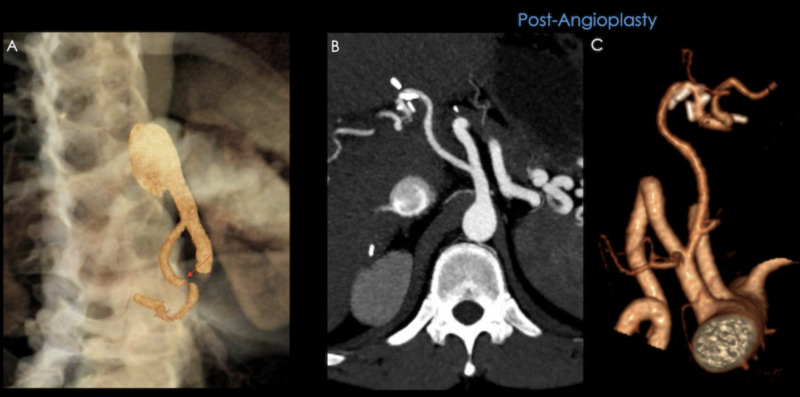
Same patient as in previous figure. A) Volume rendered image showing a site of stenosis at the extrahepatic portion of the hepatic artery. Post-angioplasty: B) Maximum intensity projection and C) Volume rendered image, showing a permeable hepatic artery with a uniform diameter.

As mentioned before indirect findings may also be seen in HAT, severe aortoiliac atherosclerosis, and reperfusion damage, within the first 48 hours following surgery [9,11]. Positive findings must be confirmed with CTA, MRA or angiography. HAS diagnosis is crucial to avoid HAT and graft failure through treatment with percutaneous angioplasty or stent placement (see Figures 13, 14).

Hepatic pseudoaneurysm

Unusual complication (<1%), mainly related to angioplasty (extrahepatic, most commonly at the site of anastomosis), graft biopsies, or focal parenchymal infections (intrahepatic) [9]. They have an asymptomatic clinical presentation; however, fistulization towards the biliary tree or digestive tract can occur and is the most feared complication. Rupture can lead to hypovolemic shock.

**Figure 15:**
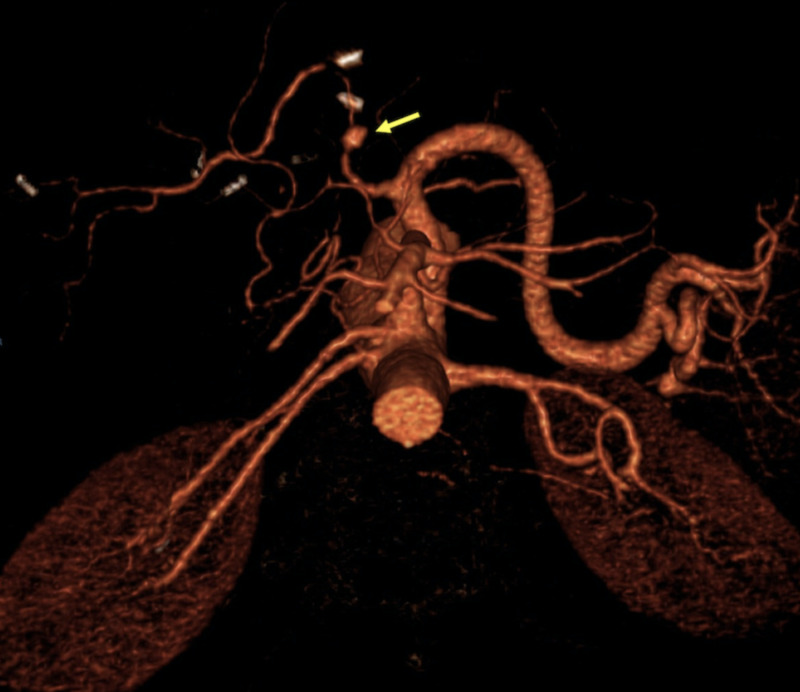
Computed tomography angiography (CTA) volume rendered image showing a saccular dilation at the pre-anastomotic portion of the hepatic artery, consistent with a pseudoaneurysm (yellow arrow).

**Figure 16:**
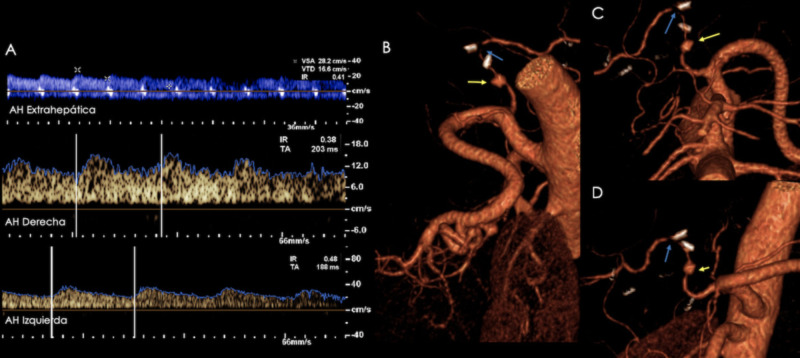
Patient with a history of orthotopic liver transplant presenting with abnormal liver function tests. A) Hepatic artery spectra (extrahepatic, right, left) showing diminished resistance indices (0.41, 0.38, and 0.48, respectively) and a "tardus et parvus" morphology. Since a site of stenosis was not identified, a CT angiography was performed due to a high suspicion of a more proximal site of stenosis. B-D) Volume rendered images showing a site of stenosis at the hepatic artery's anastomosis (blue arrows). A pseudoaneurysm proximal to the site of anastomosis was also identified (yellow arrows).

Ultrasound can show a fluid containing lesion along the hepatic artery's tract, with a color-filled interior (yin-yang sign) and a “to and fro” spectral pattern (anterograde systole and retrograde diastole) on pulse Doppler ultrasound evaluation. The lesion will enhance on CTA/MRA (see Figures 15, 16). Pseudoaneurysms may be treated with percutaneous embolization, covered stent exclusion, or surgery.

Splenic steal syndrome

It is also known as non-occlusive hepatic hypoperfusion syndrome. The reported incidence is 4.7% with 93.7% of cases occurring in the first two months [16]. There is diminished hepatic artery flow due to deviation towards a prominent splenic artery (common in patients with pre-existing portal hypertension and significant splenomegaly), resulting in hepatic hypoperfusion.

**Figure 17:**
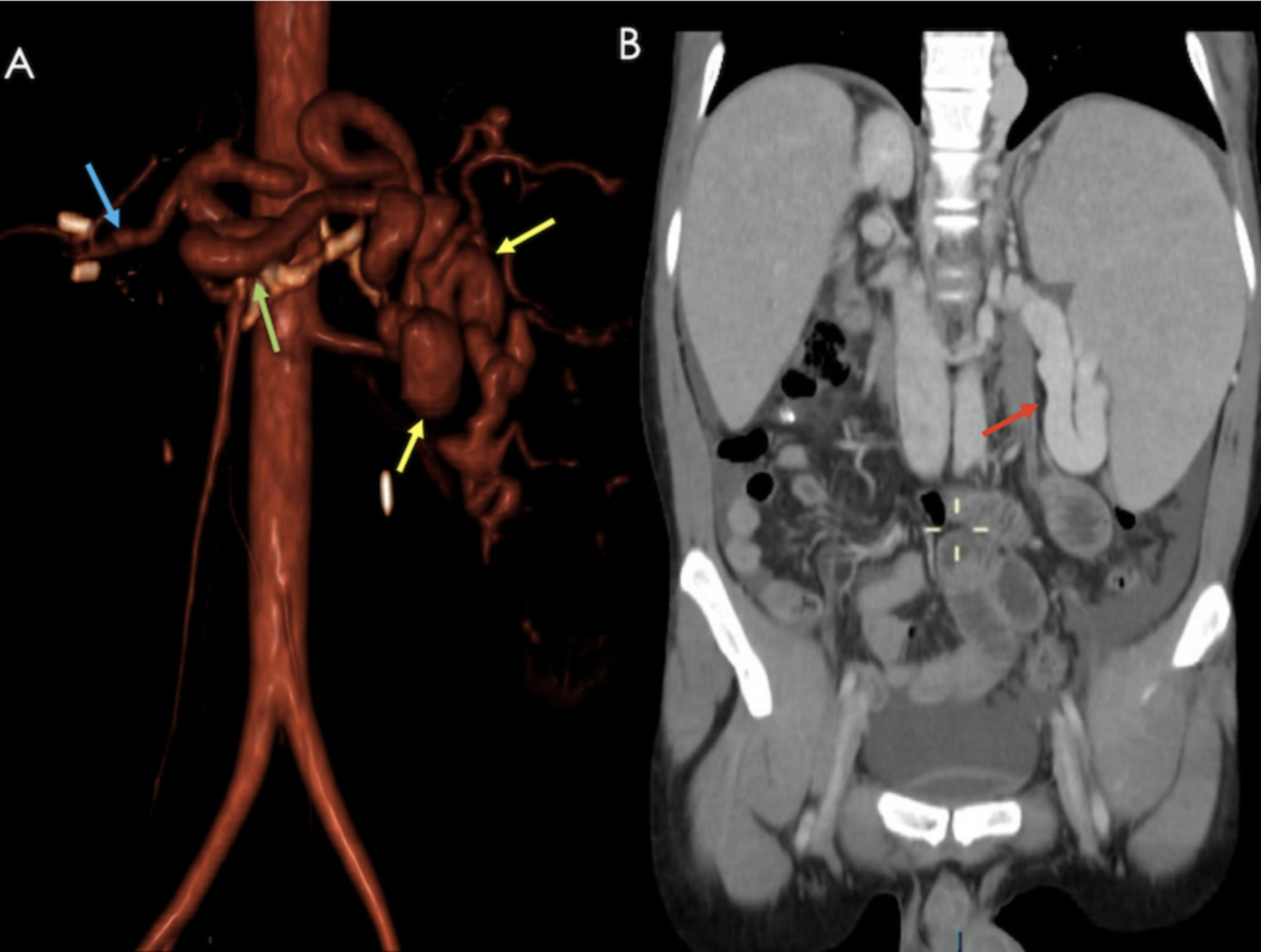
Patient presenting 27 days after a post-orthotopic liver transplant with abnormal liver function tests. A CT angiography was performed. A) Volume rendered image showing a prominent splenic artery (green arrow) with peripheral branches (yellow arrows); a permeable hepatic artery is also seen (blue arrow). B) Coronal reconstruction showing massive splenomegaly and a prominent splenic artery (red arrow).

**Figure 18:**
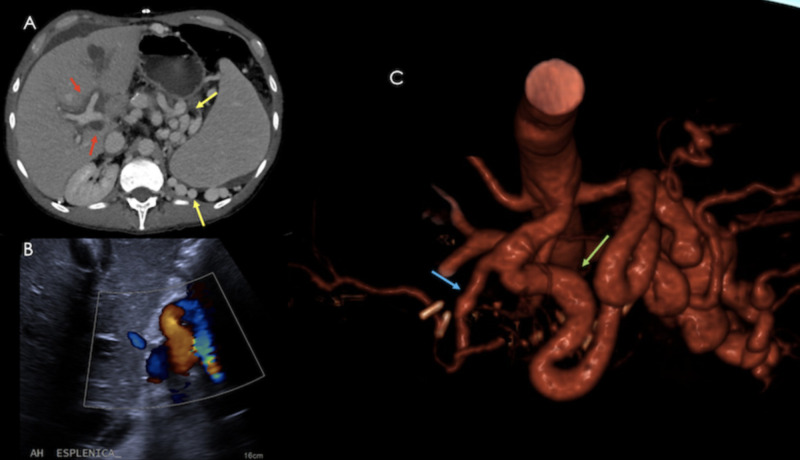
Same patient as previous figure. A) Axial contrast-enhanced CT showing periportal edema (red arrows) and prominent splenic arterial circulation (yellow arrows). B) Color Doppler US showing a prominent and tortuous splenic artery. C) Volume rendered image showing a splenic (Green) : hepatic (Blue) artery ratio >2:1. All these findings are consistent with splenic steal syndrome.

A splenic volume higher than 829cc and a splenic to hepatic artery diameter ratio >1.6 have been identified as risk factors (see Figures 17, 18) [16].

Imaging findings are non-specific, however, on angiography, flow predominance towards the splenic artery with early preferential splenic parenchymal perfusion, and portal venous flow occurring simultaneously with the splenic arterial flow before complete filling of the hepatic artery, are associated subjective findings [9,16]. Proximal splenic artery embolization is considered first-line treatment.

Portal vein complications

Portal vein complications are unusual and less common than arterial complications. They include portal vein thrombosis and stenosis.

Portal vein thrombosis

Portal vein thrombosis usually occurs at the site of anastomosis and/or extrahepatic segment, approximately one month after transplantation, with an overall incidence of 1-3% [13]. Clinical manifestations range from portal hypertension to liver failure.

Risk factors [4,9,11]:

- Hypercoagulable states

- Increased downstream resistance

- Decreased portal inflow

- Prior portal vein surgery, such as transjugular intrahepatic portosystemic shunt (TIPS) or thrombosis

- Technical problems (vessel misalignment, significant differences in calibers)

**Figure 19:**
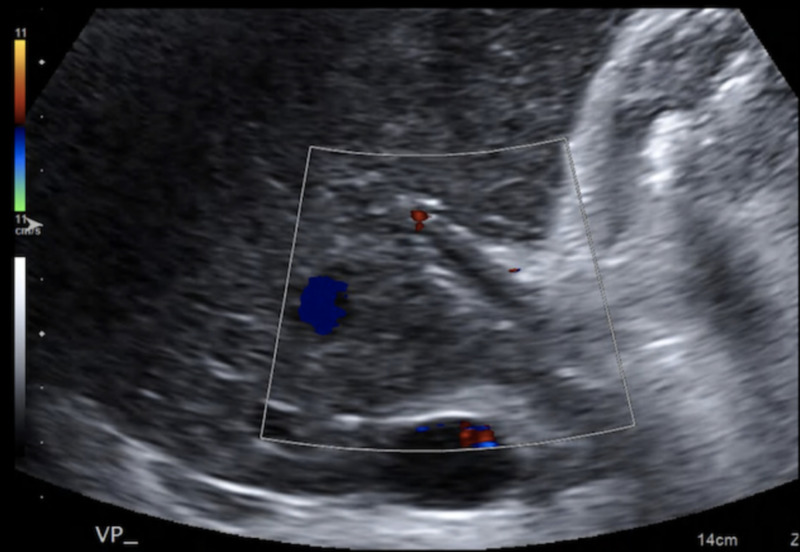
Color Doppler ultrasound (US) showing an absence of saturation of the portal vein at the porta hepatis and at its extrahepatic portion, consistent with acute thrombosis.

Color Doppler ultrasound examination demonstrates no blood flow in the portal vein (see Figure 19). A non-obstructive thrombus is identified as an anechoic (acute) or echogenic filling defect in the portal vein.

Portal vein stenosis

Portal vein stenosis usually occurs at the site of the anastomosis with a reported incidence of less than 1% [11]. Presentation within the first six months is often due to technical reasons. A more delayed presentation is frequently caused by neointimal hyperplasia [4].

Signs of portal vein stenosis on US include a focal narrowing of the main portal vein, a stenosis-to-pre-stenosis ratio >2.5-3:1, and portal vein peak systolic velocity higher than 125 cm/s [9,12,13].

**Figure 20:**
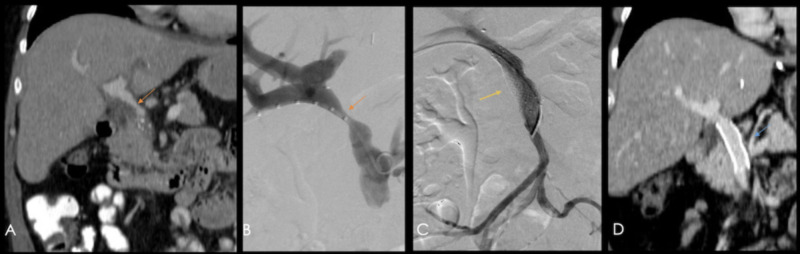
60-year-old patient with a history of orthotopic liver transplant due to Barcelona clinic liver cancer (BCLC) A hepatocellular carcinoma. A) Coronal reconstruction of a contrast-enhanced CT showing a focal zone of diminished caliber in the portal vein (orange arrow) at the porta hepatis and at its extrahepatic portion. B) Portography showings a focal decrease in caliber (orange arrow). C) A portal stent (yellow arrow) was placed during the same procedure. D) Coronal reconstruction of a follow-up contrast-enhanced CT performed four years after stent placement, showing adequate permeability (blue arrow).

Treatment options include catheter-guided angioplasty and covered stent placement (see Figure 20).

Inferior vena cava and hepatic veins complications

Inferior vena cava and hepatic veins complications are very rare with an estimated incidence of 1-2%, usually presenting at the site of anastomosis or due to extrinsic compressions (graft swelling, hematoma or fluid collections) [17].

Risk factors [10]:

- A discrepancy between donor and recipient vessels

- Suprahepatic caval kinking

**Figure 21:**
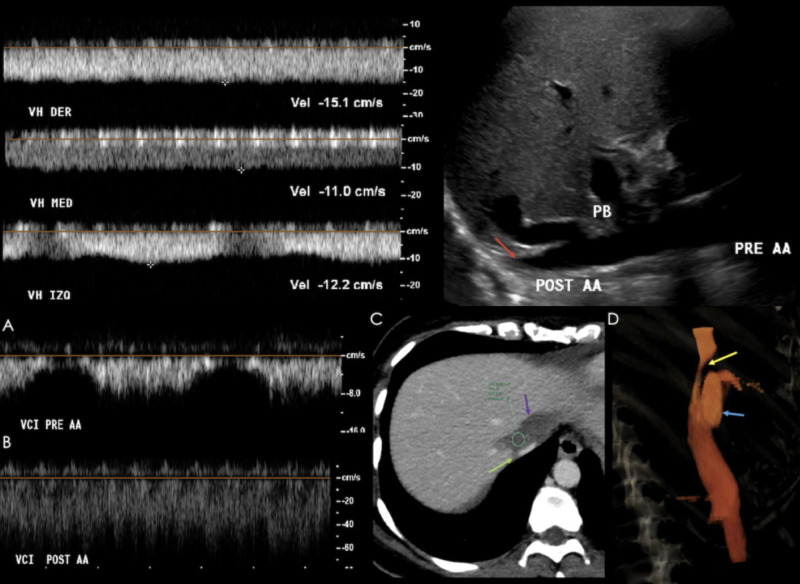
42-year-old female in her 21st day following orthotopic liver transplant. Right upper images: anterograde spectra showing a loss of phasicity and pulsatility of the right, middle, and left hepatic veins. Left upper image: Grey-scale ultrasound (US) showing "piggy-back" changes at the site of anastomosis, with a zone of stenosis (red arrow) at the suprahepatic vena cava. A, B) Pre- and post-anastomosis IVC spectra, showing a significant decrease in velocity, with a post-stenotic : pre-stenotic velocity ratio >4:1. C) Axial contrast-enhanced CT showing a focal site of stenosis (green arrow) due to extrinsic compression from a hematoma (purple arrow). D) Volume rendered image showing the site extrinsic compression (yellow arrow) at the suprahepatic IVC, and "piggy-back" changes (blue arrow).

In the case of inferior vena cava stenosis, Doppler ultrasound demonstrates loss of phasicity and pulsatility of the hepatic veins (loss of transmission of cardiac pulsations), a reduction of caliber at the site of stenosis with pre-stenotic dilation, a stenotic-to-pre-stenotic velocity ratio >3-4:1, and a hepatic vein pulsatility index of less than 0.45 (outflow stenosis) (see Figure 21) [3, 13, 16].

**Figure 22:**
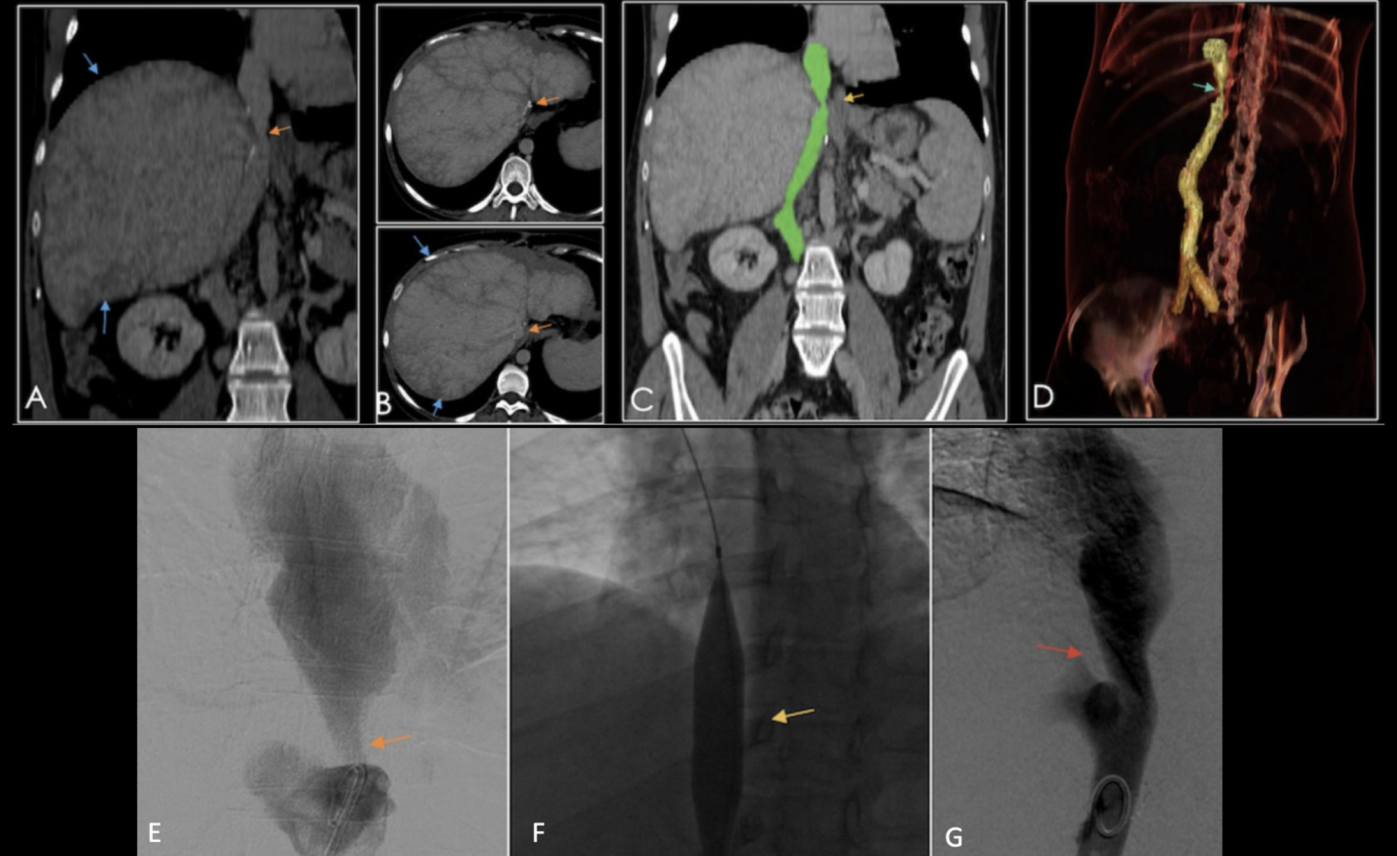
26-year-old patient with a history of orthotopic liver transplant due to secondary biliary cirrhosis, presenting with bilateral leg edema. A) Coronal reconstruction and B) axial contrast-enhanced CT showing perfusion defects in the hepatic parenchyma (blue arrows), and vena cava stenosis (orange arrows). C & D) Coronal reconstruction and volume rendered imaging showing the site of vena cava stenosis (yellow and blue arrow, respectively). E) Venography showing vena cava stenosis (orange arrow) after a piggy-back anastomosis. F) A balloon angioplasty (yellow arrow) was subsequently performed, achieving adequate permeability in follow-up studies (red arrow in G).

Knowing the surgical technique used for the IVC anastomosis is essential. The “piggyback” anastomosis is vulnerable to several complications such as hemorrhage secondary to dehiscence of the cavo-caval anastomosis, and Budd-Chiari syndrome due to inadequate venous drainage (see Figure 22).

Contrast-enhanced cross-sectional modalities are used to confirm findings (focal reduction in diameter, inferior vena cava filling defects, and mosaic patterns of perfusion) (see Figure 22).

Biliary complications

Biliary complications are the second most common cause of graft dysfunction, after rejection [18]. They have an overall incidence of 5-25% (most common in patients following a right lobe living donor transplantation) and occur during the first post-surgical month [9, 10].

Biliary complications are consequences of HAT and HAS in 90% and 70% of cases, respectively.

Biliary complications include strictures, obstructions, fistulas, leaks, and recurrent biliary disease.

MR cholangiography is the best imaging modality for the evaluation of the biliary tree.

Bile leaks:

Bile leaks occur in approximately 5% of transplanted patients in the early post-surgical period (70% within the first month), most commonly at the T-tube exit site and biliary anastomosis [11]. Bile leakage at the distal or intrahepatic bile ducts is usually indicative of biliary necrosis secondary to HAT or HAS and generally requires re-transplantation [9]. Bile may leak into the peritoneal cavity or form a bilioma (perihepatic bile collection).

Cholangiography is the most precise imaging technique; however, endoscopic retrograde cholangiopancreatography and cholescintigraphy are other sensitive and specific techniques [13]. Bile leaks appear as a "double duct” sign of contrast extravasation along the T tube into the peritoneum on cholangiography or can form a bile collection [12]. US is non-specific as ascites, hematomas, and abscesses can mimic bile leakage.

Biliary obstruction and stenosis

Biliary obstruction is the most common biliary complication, frequently caused by stenosis at the site of anastomosis, due to fibrotic proliferation. Non-anastomotic strictures occur in 8% of transplants and should raise concern for biliary ischemia (HAT or HAS). Other causes of non-anastomotic strictures include pre-transplantation biliary disease and infection [11].

In the event of diffuse ductal dilations, papillary dyskinesia due to devascularization/denervation of Vater’s ampulla should be ruled out.

Biliary stenosis is not always evident on ultrasound in hepatic transplant patients. If there is a high grade of clinical suspicion, an MR cholangiography, endoscopic retrograde cholangiopancreatography (ERCP) or cholangiography should be performed. Cholangiography and MR cholangiography have shown a sensitivity and specificity of 87% and 92%, respectively.

Biliary stones occur in 5.7% of post-transplanted patients [13]. MR cholangiography is specific in distinguishing them from cast-like appearance and sludge.

Neoplasms

Transplanted patients are at higher risk of developing neoplasms. Transplanted hepatic patients have a 100% greater risk of developing neoplastic lesions due to immunosuppression (higher incidence than in kidney transplants) [9,11]. Therefore, the presence of a solid intraparenchymal mass should always raise concern for malignancy. The extrahepatic disease is more common than intrahepatic involvement. B-cell or squamous cell cutaneous lesions, Kaposi’s sarcoma, and particularly non-Hodgkin lymphoma (Epstein-Barr viral infection) are the most commonly occurring neoplasms [12]. Most cancers develop two to six years after transplantation [9].

When the Milan Criteria (unique lesion less than 5 cm in diameter, or a maximum of three lesions no more than 3 cm in diameter) are applied on patients with a prior history of HCC, the reported recurrence rate is less than 10% and the survival rate increases up to 70-75% [12].

Fluid collections

Post-transplant fluid collections are commonly observed in areas of anastomosis and Morrison’s pouch. Perihepatic hematomas, seromas, and right-sided pleural effusions are most commonly seen. These collections usually disappear within the first post-surgical weeks. They can rarely compress vascular structures, such as the portal vein or IVC. Hematomas are avascular and echogenic during the acute phase, becoming more hypoechoic to anechoic over time [9].

Post-transplanted liver follow-up algorithm

**Figure 23:**
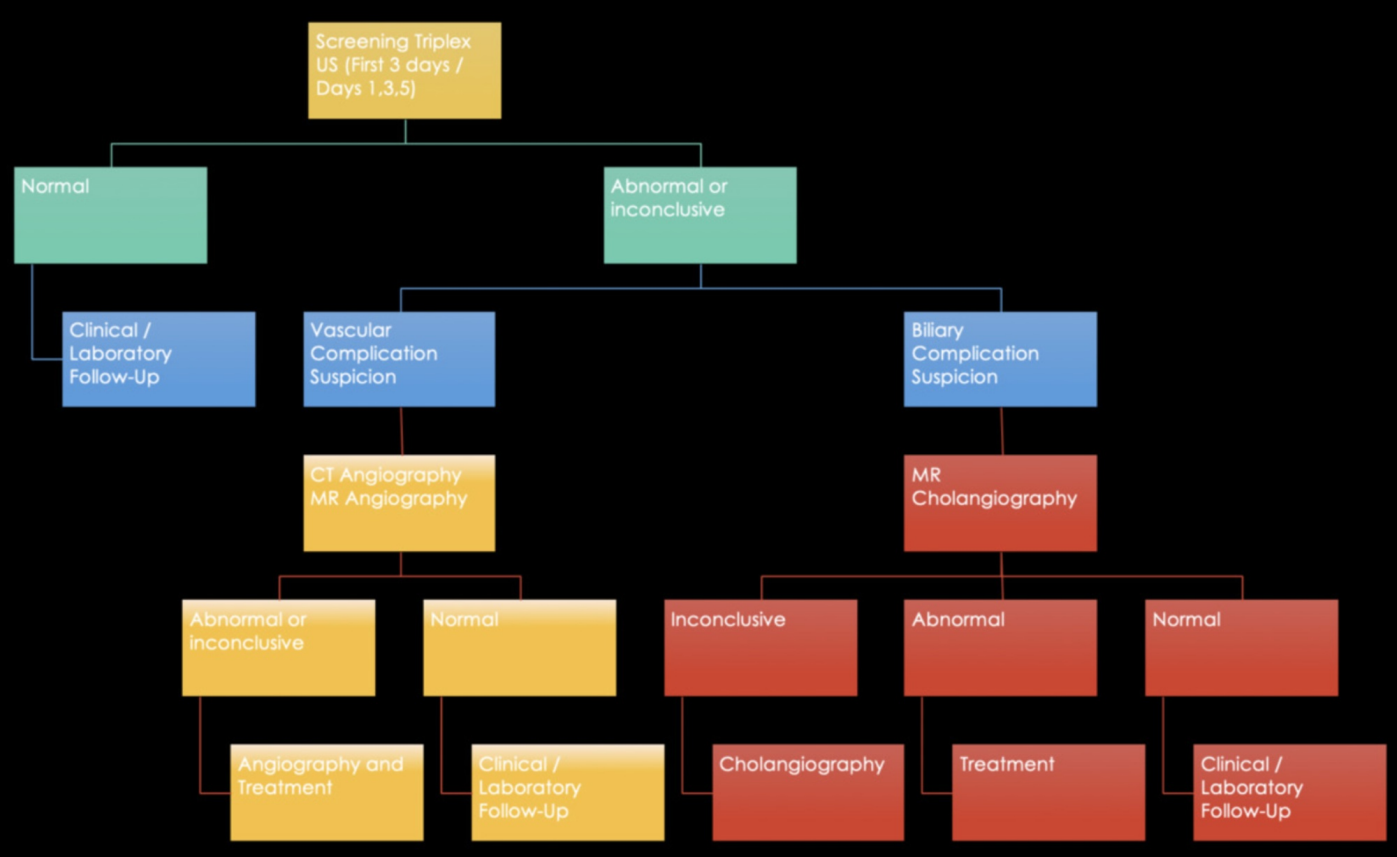
Post-transplanted liver follow-up algorithm.

A follow-up algorithm is proposed to help guide imaging evaluation in graft recipients (see Figure 23).

Please note that all the figures incorporated in the article have been presented at the European Congress of Radiology 2020 by the author [19].

## Conclusions

Imaging studies play a fundamental role in the diagnosis of liver transplant complications and patients' follow-up. Doppler ultrasonography, a non-invasive and low-cost imaging modality, is the first-line study during post-surgical evaluation for graft dysfunction and screening of liver transplant patients. It has a high sensitivity in the diagnosis of vascular and biliary complications. Ultrasonography, CT, and MRI complement each other. It is essential to differentiate expected from abnormal findings to layout an appropriate plan of action. Radiologists must be familiarized with transplant complications findings. An imaging multimodality approach is endorsed for adequate diagnosis and treatment planning. Timely detection of complications is vital for prompt treatment and to maintain graft function. It is important to remember that if there is suspicion of rejection, different imaging modalities have a limited role.
